# Retained After-Birth in Cows1Read before the Twelfth Annual Meeting of the New York State Veterinary Society, Brooklyn, September 9 and 10, 1902.

**Published:** 1902-10

**Authors:** W. L. Williams

**Affiliations:** Ithaca, N. Y.


### RETAINED AFTER-BIRTH IN COWS.[1]

By W. L. Williams, V.S.,
ITHACA, N. Y.

We cannot absolutely define in point of time what constitutes retention of the secundines, since what we may deem delayed expulsion in one case may readily be regarded as perfectly normal in another, although the duration of time elapsing between parturition and expulsion of fœtal membranes be the same in each. A heifer usually expels the fœtal envelopes more tardily than the adult cow, and the expulsion of membranes is usually more prompt where the fœtus has been carried to full term than in case of premature birth or abortion.

Retention is, therefore, a relative term, and signifies a delay in the expulsion of the fœtal membranes of such a duration or character as to constitute a menace to the health of the patient. This period is usually reached in from twenty-four to forty-eight hours after calving, but danger may occur earlier and is not infrequently delayed later.

The symptoms of retention are usually too evident to be mistaken, consisting of the protrusion of portions of the secundines from the vulva, with a variable discharge, and symptoms of local and constitutional disturbances of great diversity according to conditions.

In some cases there is complete retention, no parts being visible, when the disease may be expressed by vulvovaginal discharge; or this may fail, and especially where only a small portion of the membranes remain there may be present only symptoms of septicæmia, and our attention is called to the uterus chiefly through the history of recent parturition without other evidence of disease, and suspicion is set at rest by manual exploration of the genital passages.

In rare cases we meet with eversion of the uterus or vagina and with tetanus as complications.

The causes of retention of the fœtal membranes will not be fully known until the method of detachment and expulsion is understood. We do not know why birth takes place at a certain time, nor why it should be almost immediately followed or accompanied or even preceded by expulsion of the fœtal membranes. Writers on vet-

[1] Read before the Twelfth Annual Meeting of the New York State Veterinary Society, Brooklyn, September 9 and 10, 1902.

erinary obstetrics generally recognize two chief classes of causes for retention—inflammatory adhesion of the fœtal to the maternal placenta, and tardy involution of the uterus.

The first is largely theoretical and not well verified by clinical evidence. The possibility of such a state may well be admitted, but we find no convincing clinical record to such effect. The condition probably occurs but rarely; the cases so termed being, in our judgment, generally errors in diagnosis. The real condition is a placentitis, causing tumefaction of the maternal placenta, resulting in incarceration of the tufts of the fœtal placentæ, rendering them undetachable.

Tardy involution of the uterus, if not the prevailing cause, is certainly almost a constant condition. But we need not accept the presence of this state as the cause of retention. If we place a pack of gauze in the uterus and retain it there, the organ relaxes, becomes flaccid, paretic, and it would seem that the presence of the after-birth might also exert a retarding influence on involution. In some cases we note further that clinically the involution may be fairly prompt, but a few maternal cotyledons, being inflamed, incarcerate and firmly retain some of the fœtal placenta.

Neither is complete uterine involution essential to expulsion of the secundines, for we not rarely see in delayed labor the fœtal membranes detached in part or wholly, and very rarely even expelled prior to the expulsion of the fœtus. It would consequently appear that tardy involution may be rather coincidental or a parallel condition to rather than a fundamental cause of retained fœtal envelopes, and that the real cause may be best sought in a general interruption of uterine functions, which includes involution as one of its most prominent activities.

We might compare these complex uterine functions to those taking place in the alimentary tract during the process of digestion, where muscular contraction of the walls of the digestive tube is constant, but in itself is totally inadequate to digest aliment, certain glandular and other activities being essential parts of the function.

The thought that uterine contraction suffices to detach the fœtal membranes might be accepted in some animals, but with the cow we have no knowledge of any muscular elements in the maternal cotyledons beyond those of the bloodvessels, and it is difficult to understand how the contraction of the uterine walls can affect the attachments existing between the maternal cotyledon and the fœtal placenta. At times, moreover, the contraction of the uterine walls constitutes the chief cause of the retention.

We feel warranted in concluding from the foregoing observations that uterine involution plays only a subsidiary part in the expulsion of the fœtal membranes.

The causes of tardy or incomplete involution along with general derangement of the uterine functions may be very numerous, among the most prominent of which are debility of the patient, exhausting labor, premature labor or abortion, placentitis, metritis, wounds of the uterus, overstretching of uterine walls by hydrops amnii, etc., parturient paresis.

The treatment of retained membranes should be based upon a rational understanding of the causes at work in each given case. For example, in parturient paresis it will usually suffice to pay general attention to the paresis, and, upon its yielding, the placenta will ordinarily be expelled.

When due to debility of the animal, tonics and stimulants are indicated; while in case of wound, metritis, placentitis, etc., where infection plays the chief rôle, effective means of disinfection are called for, and in fact we might say that the ultimate object of all plans of treatment of retained fœtal membranes is to bring about disinfection of the uterus and vagina.

Before these can be undertaken rationally, careful manual exploration of the uterine cavity is essential. Most writers and practitioners have greatly overdrawn the repulsiveness and dangers of such examination. Properly conducted, it is not very disagreeable nor highly dangerous. Before inserting the hand into the uterus with its putrid contents, the organ should be thoroughly flushed out with five-tenths gallon or more of a tepid disinfecting solution of say 1 per cent. of lysol. The operator's hands and arms should be thoroughly disinfected with a carefully selected preparation. Wounds or abrasions on the hands or arms are always dangerous, and are, perhaps, best guarded by cauterizing the parts and then saturating the cautery scab with a reliable disinfectant.

Most infections probably occur through the hair follicles and sweat glands, and ordinary disinfectants do not penetrate these. We have been led to conclude by our observations that operators who sweat freely most readily become infected. Two plans for avoiding infection through the hair follicles and sweat glands suggest themselves.

Astringent antiseptics like permanganate of potash or nitric acid may tend to constringe or occlude the glandular openings in the skin, or disinfectants having the power of penetrating through sebum, etc., into the cutaneous glands and being deposited therein in a solid state like an alcoholic solution of corrosive sublimate, the tincture of iodine, etc.

Examination may reveal a variety of conditions, which we may consider in three chief groups:

1. The after-birth may be wholly detached and merely retained by contraction of the cervix or other mechanical impediment, or may be partly or wholly attached in such a manner as to allow of its ready detachment, the fœtal tufts separating easily from the maternal cotyledons, none of the fœtal papillæ remaining behind in the cotyledon when mechanical separation is properly attempted. The only handling required is the careful manual detachment of the fœtal from the maternal cotyledons, the withdrawal of the secundines from the uterus, and thorough flushing of the uterine cavity with a mild disinfectant solution.

2. The membranes may be firmly attached to the maternal cotyledons, and any attempt at separation will result in wounding the maternal cotyledons, with attendant hemorrhages and great increase of danger of infection; or the tufts or papillæ of the fœtal cotyledons break off and remain in the maternal placenta, the latter closing over them, rendering their presence a greater menace in this condition than if the entire membranes had been left undisturbed. In the absence of fever or marked irritation it is bad surgery to attempt manual removal. It is advisable in such cases to stimulate the uterine functions and delay putrefaction of the membranes.

A great number of drugs have been recommended to be given in such cases. Ergot has been lauded by some; probably more practitioners have favored the use of stimulants and carminatives, such as various vinous distilled and malt liquors with ginger, fenugreek, anise, fennel, etc. The value of general stimulants and carminatives in favoring expulsion is doubted by few, but the reliance to be placed in these is not equally regarded by various practitioners. Some rely on these almost wholly in nearly all cases. Injections of cold water are favored by some as stimulating uterine contraction. Injections of warm antiseptic solutions in large quantities have proven highly valuable in our hands. They arrest putrefaction of the membranes when used freely twice a day, and hasten the detachment by stimulating normal uterine functions. Internal medication as suggested above may well be combined with this method. Finally, when detachable treat as under No. 1.

3. Placentitis may occur within a few hours after parturition; the cotyledons are tumefied, much enlarged, firm, and the tufts of the fœtal membranes are incarcerated in the maternal cotyledons and held as if in a vise. These incarcerated fœtal papillæ are putrefying, causing septicæmia and high fever. Such conditions brook no delay and call for the most effective disinfection possible. Tearing away the fœtal from the maternal cotyledons only adds to the danger, already great; the injection into the uterus of large volumes of disinfectants can exert but feeble influence on the disease processes going on deep within the enlarged cotyledons. As a rule, the cotyledons in these cases can be detached without maternal hemorrhage, and if so they should all be carefully pinched or twisted off and the uterus flushed out with disinfectants. Fleming mentions this process as having been done by empirics. It is equally proper and salutary when done by the educated veterinarian. It corresponds in its effects to the curetting of the placenta of women. We must look upon the maternal cotyledon in this case as a vast diseased surface, in contact everywhere with the necrotic, putrefying papillæ of the fœtal placenta. If we remove the maternal cotyledon at its neck, we leave a wound area probably less than $\frac{1}{1000}$ of the area of contact between the maternal and fœtal cotyledons, and in a tissue in a state of health more capable of resisting infection. If we delay action and the animal survive, these cotyledons become necrotic, drop off, and are eventually expelled. We have observed the temperature drop 3 or 4 degrees in a few hours after removal of the cotyledons and antiseptic flushing of the uterus. The operation is advised in proper cases only. We have observed fatal hemorrhages where such an operation was attempted by a charlatan almost immediately after calving where no action was really indicated.

To recapitulate, we meet with three chief conditions calling for as many distinct plans of handling:

1. Cases where the after-birth is readily or completely detachable, in which it should be removed manually and the uterus flushed out with antiseptics.

2. Cases where the after-birth is not detachable without violence, in which cases alcoholics, carminatives, and stimulants, internally, with repeated uterine injections of warm antiseptic solutions are indicated.

3. Placentitis, in which removal of the cotyledons is to be recommended.

In all cases of prolonged manipulation, frequent injections of large volumes of antiseptics are of the highest importance, modifying greatly the repulsiveness of the operation and reducing to the lowest degree the danger of infection both to operator and patient, facilitating the operation by washing away the débris and by stimulating uterine contractions, which serve to bring the farther parts of the uterus more readily within the operator's reach.

In addition a large volume of antiseptic solution should be kept at hand, and the operator should thoroughly wash his hands and arms in this at intervals of a few minutes.

When the work is done any fetor remaining on the operator's hands and arms can be removed by bathing in a hot solution of permanganate of potash and the stain therefrom removed by washing in a strong solution of oxalic acid.

It is needless to say that the clothing to be worn during the operation should be of a character readily sterilized by boiling, and the less worn the better as far as compatible with temperature and environment.

